# Application of smart devices in investigating the effects of air pollution on atrial fibrillation onset

**DOI:** 10.1038/s41746-023-00788-w

**Published:** 2023-03-14

**Authors:** Cong Liu, Meihui Tai, Jialu Hu, Xinlei Zhu, Weidong Wang, Yutao Guo, Haidong Kan, Renjie Chen

**Affiliations:** 1 School of Public Health, Shanghai Institute of Infectious Disease and Biosecurity, Key Lab of Public Health Safety of the Ministry of Education and NHC Key Lab of Health Technology Assessment, Fudan University, Shanghai, 200032 China; 2 Pulmonary Vessel and Thrombotic Disease, Sixth Medical Center, Chinese PLA General Hospital, Beijing, 100048 China; 3 Department of Cardiology, Zhongshan Hospital Affiliated to Fudan University, Shanghai, 200032 China; 4 Department of Biostatistics and Epidemiology, School of Public Health and Health Sciences, University of Massachusetts Amherst, Amherst, MA 01003 USA; 5 Children’s Hospital of Fudan University, National Center for Children’s Health, Shanghai, 201102 China

**Keywords:** Atrial fibrillation, Environmental impact

## Abstract

Few studies have examined the link between short-term exposure to air pollutants and atrial fibrillation (AF) episodes. This study aims to examine the association of hourly criteria air pollutants with AF episodes. We employ a smart device-based photoplethysmography technology to screen AF from 2018 to 2021. Hourly concentrations of six criteria air pollutants are matched to the onset hour of AF for each participant. We adopt a time-stratified case-crossover design to capture the acute effects of air pollutants on AF episodes, using conditional logistic regression models. Subgroup analyses are conducted by age, gender, and season. A total of 11,906 episodes of AF are identified in 2976 participants from 288 Chinese cities. Generally, the strongest associations of air pollutants are present at lag 18–24 h, with positive and linear exposure-response relationships. For an interquartile range increase in inhalable particles, fine particles, nitrogen dioxide, and carbon monoxide, the odds ratio (OR) of AF is 1.19 [95% confidential interval (CI): 1.03, 1.37], 1.38 (95%CI: 1.14, 1.67), 1.60 (95%CI: 1.16, 2.20) and 1.48 (95%CI: 1.19, 1.84), respectively. The estimates are robust to the adjustment of co-pollutants, and they are larger in females, older people, and in cold seasons. There are insignificant associations for sulfur dioxide and ozone. This nationwide case-crossover study demonstrates robust evidence of significant associations between hourly exposure to air pollutants and the onset of AF episodes, which underscores the importance of ongoing efforts to further improve air quality as an effective target for AF prevention.

## Introduction

Cardiovascular disease (CVD) is the leading cause of death worldwide, posing the greatest threat to the global disease burden. According to the 2019 Global Burden of Disease study (GBD 2019), CVD was the top-ranked cause of disability-adjusted life years for adults and the elderly^1^. Atrial fibrillation (AF) is the most common arrhythmia, which can progressively lead to blood clots, stroke, heart failure, and other cardiovascular/cerebrovascular complications^2^. There are estimated 37.6 million (95% uncertainty interval: 32.5 to 42.6 million) individuals diagnosed with AF, according to a report from the GBD 2017 study^3^. A clinical diagnosis of AF generally requires clinical pulse palpation and 12-lead electrocardiogram (ECG)^4^, leading to difficulties in screening, monitoring, and managing AF. The low diagnostic rate remains to be a major challenge in current management for patients with suspected AF. Besides, early detection of recurrent AF episodes is also an important clinical practice to alleviate the development of severe arrhythmia events or comorbidities. With recent advances in mobile and wearable devices, new technologies such as single-lead ECG and photoplethysmography (PPG) have provided possible solutions for screening or early detecting AF in general populations or susceptible subgroups^5–7^.

Ambient air pollution is a well-established risk factor for CVD^8^. Numerous epidemiological studies have linked ambient air pollutants with increased mortality and morbidity from CVD^9^. Among criteria air pollutants, particulate matter (PM), including inhalable particles (PM_10_) and fine particles (PM_2.5_), have been extensively linked with CVD; while the effects of nitrogen dioxide (NO_2_), sulfur dioxide (SO_2_), carbon monoxide (CO), and ozone (O_3_) on CVD were less investigated and the results were more mixed. For the acute effects of air pollution on AF, most previous studies were of time-series study design, which utilized daily numbers of hospitalizations and outpatient or emergency-room visits, which cannot account for individual-level confounders. Besides, most previous studies focused on one or very few air pollutants, resulting in potential selection bias and creating difficulty in comparing the effects of various air pollutants. Furthermore, previous studies have mostly examined the association at the daily level, which could not fully capture the sub-daily time-lag effects of air pollution^10^. Most importantly, almost all previous studies relied on medical records to identify an AF event, which may have missed a significant amount of paroxysmal or occult AF episodes. A PPG-based screening technology based on the smart device has been validated in our prior studies with a 91.6% positive predictive value of AF. Taking these aspects into account, this smart screening technology is promising in epidemiological studies linking air pollution and AF onset^11^.

As a developing country with the largest population, China faces a tremendous disease burden of AF. According to a recent national survey from 2020 to 2021, the prevalence of AF was 1.6% in the Chinese adult population^12^. Meanwhile, China has one of the highest air pollution levels in the world, especially for particulate air pollution^13^. Therefore, we designed this case-crossover study in China to evaluate the associations between hourly concentrations of all criteria air pollutants and the real-time onset of AF episodes detected from smart devices.

The findings reveal positive and linear exposure-response relationships of hourly exposure to multiple air pollutants with AF, with the largest estimates at lag 18–24 h. These associations keep robust even after the adjustment of co-pollutants, and we observe larger estimates among females, older people, and during cool seasons. This nationwide case-crossover study uses smart devices to screen AF episodes, and we find significant associations between hourly air pollutants and AF onsets, which highlights the importance of ongoing efforts to improve air quality as an effective target for AF prevention.

## Results

### Descriptive statistics

Table 1 summarizes the descriptive statistics on the included subjects and AF episodes. We identified a total of 11,906 AF episodes with 40,551 controls (3.4/1) in 2976 participants during the study period from 2018 to 2021, covering 288 cities in China (Supplementary Fig. 2). For AF episodes, males accounted for a significantly larger proportion (85.9%) than females (14.1%), and there were more cases in the warm season (54.8%) compared with the cold season (45.2%). Notably, our study observed more AF episodes in individuals with age <60 years (59.7%). Meanwhile, the wearing rate was also higher in the subgroup of 22–39 years old (54.5%) than in the subgroup of 65 years or more (32.4%).

**Table 1:** Descriptive statistics on study participants and atrial fibrillation episodes.

Variables	Participants (*N*, %)	Episodes (*N*, %)
Atrial fibrillation	2976	11,906
Controls	2976	40,551
Sex		
Males	2520 (84.7%)	10,229 (85.9%)
Females	456 (15.3%)	1677 (14.1%)
Age		
<60	1803 (60.6%)	7135 (59.9%)
≥60	1173 (39.4%)	4741 (40.1%)
Season^a^		
Cold	1311 (44.1%)	5376 (45.2%)
Warm	1665 (55.9%)	6530 (54.8%)

^a^Season: Warm season, April to September; Cold season, October to March.

Supplementary Table 1 summarizes the environmental data averaged 24 h prior to the onset hour of AF or its controls. The average exposure levels (over 0–24 h) of PM_10_, PM_2.5_, and NO_2_ during case periods were 60.5, 34.2 μg/m^3^, and 30.0 μg/m^3^, respectively, which were slightly higher than those during control periods. The average exposure levels (0–24 h) of SO_2_, O_3_, and CO were quite similar between case periods and control periods. The exposure differences between case and control periods were generally larger at lag 18–24 h than other lag intervals (Supplementary Table 2). The interquartile ranges (IQRs) of PM_10_, PM_2.5_, NO_2_, SO_2_, O_3_, and CO during 24 h prior to the onset hour of AF were 45.3, 25.8, 22.0, 5.8, 40.6, and 0.4 mg/m^3^, respectively. There were no apparent differences between temperature and relative humidity during the case and control periods. The Spearman correlations between air pollutants and metrological factors were provided in Supplementary Table 3. PM_2.5_ was strongly correlated with PM_10_ (r_s_ = 0.84), moderately correlated with NO_2_ (r_s_ = 0.57), SO_2_ (r_s_ = 0.34), and CO (r_s_ = 0.59), and negatively correlated with O_3_ and meteorological factors.

### Regression results

**Fig. 1:**
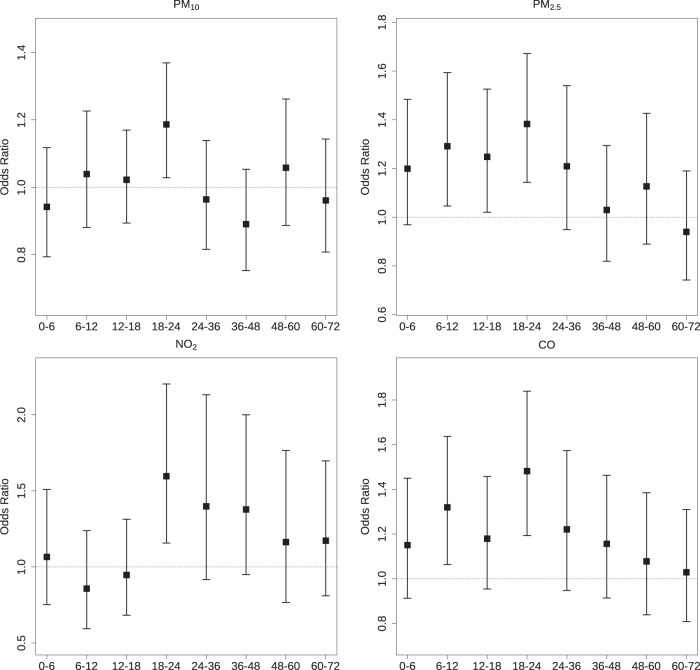
Odds ratios of atrial fibrillation associated with an interquartile range increase in air pollutant concentrations on different lag intervals. Abbreviations and interquartile range concentrations as in Table 2. Lags hours, e.g., Lag 0–6, the moving average concentrations of the current to the previous 6 h; Lag 6–12, the moving average concentrations of the previous 7 to the previous 12 h. Error bars are defined as standard deviation (s.d.).

Figure 1 presents the odds ratios (ORs) of AF episodes associated with an IQR increase of air pollutant concentrations on different lag intervals. For PM_2.5_, we observed significant and positive associations that occurred at lag 0–6 h and lasted till lag 18–24 h, whereas the estimates turned smaller and non-significant after lag 24–36 h. The associations of NO_2_ occurred later but persisted longer, as we found significant but decreasing estimates from lag 18–24 h to lag 60–72 h. There were also significant associations of CO on lag 6–12 h, lag 12–18 h, and lag 18–24 h, and of PM_10_ on lag 18–24 h. Overall, lag 18–24 h generally yielded the largest estimates for all air pollutants among all lag intervals. Thus, lag 18–24 h was used as the main lag to present results for subsequent analyses. For an IQR increase in PM_10_, PM_2.5_, NO_2_, and CO, the corresponding ORs of AF onset were 1.19 [95% confidential interval (CI): 1.03, 1.37], 1.38 (95%CI: 1.14, 1.67), 1.60 (95%CI: 1.16, 2.20) and 1.48 (95%CI: 1.19, 1.84). Supplementary Fig. 3 summarizes the lag patterns for SO_2_ and O_3_, and no significant associations were observed at any lag intervals.

**Fig. 2:**
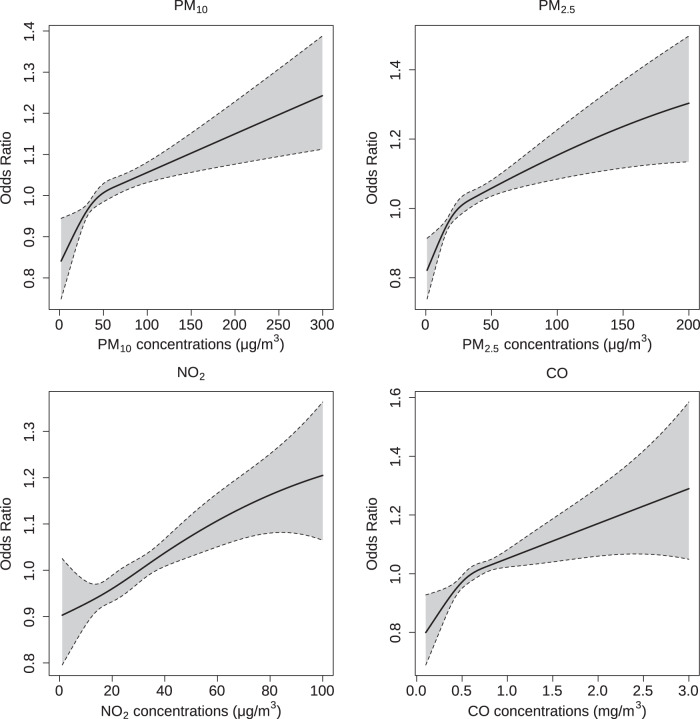
Exposure-response relationship curves between air pollutant concentrations and atrial fibrillation. The associations were presented as the odds ratio of atrial fibrillation associated with each unit increase in air pollutant concentrations at lag 18–24 h. The black lines were mean estimates and the shaded areas were 95% confidence intervals. Abbreviations as in Table 2.

Figure 2 shows the exposure-response relationship curves for four air pollutants and AF at a lag of 18–24 h. For PM_2.5_ and PM_10_, the curves were consistently increasing with higher concentrations, with a steeper slope in a concentration lower than 40 μg/m^3^. The curve for CO had a similar pattern with a slightly larger slope below 0.5 mg/m^3^. The relationship between NO_2_ and AF were almost linear and kept monotonically increasing with wider CIs at higher concentrations. The curve for SO_2_ had a positive slope but turned flat with wide CIs, while there was a decreasing but non-significant curve for O_3_ (Supplementary Fig. 4).

**Fig. 3:**
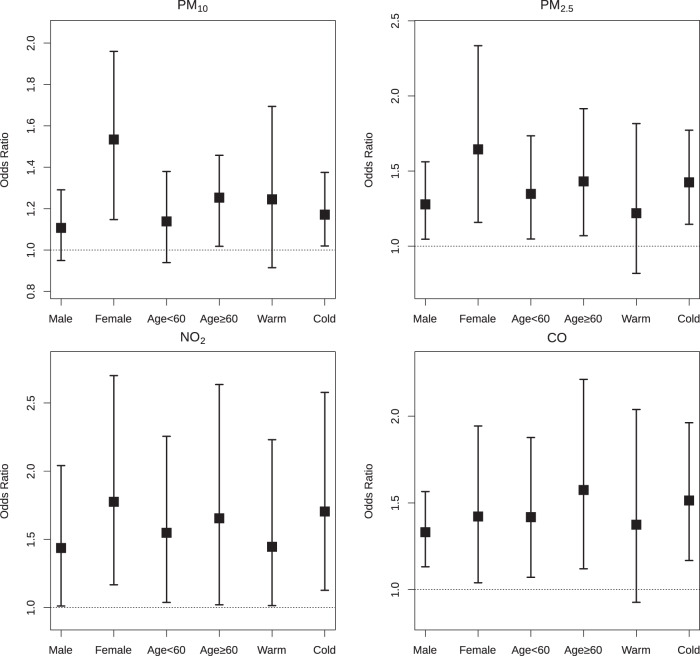
Odds ratios of atrial fibrillation associated with an interquartile range increase in air pollutant concentrations at lag 18–24 h, stratified by gender, age, and season. Abbreviations and interquartile range concentrations as in Table 2. Warm season, April to September; Cold season, October to March. Error bars are defined as standard deviation (s.d.).

Figure 3 illustrates the risk estimates in stratified analyses. The effects of air pollutants on AF were apparently larger in females than males, though the between-group differences were not statistically significant. Taking PM_2.5_ for an example, the OR per IQR increase was 1.28 (95%CI: 1.05, 1.56) in males, and the OR in females was 1.64 (95%CI: 1.16, 2.33), with an insignificant between-group difference (*P* value = 0.09). There were slightly stronger associations of AF with all air pollutants in the older group (≥60 years), and larger effects in the cold season (except for PM_10_). There were similar trends in subgroup-specific estimates for SO_2_ and O_3_ (Supplementary Fig. 5). The between-group differences were also insignificant for age subgroups and seasons.

The estimated associations in two-pollutant models are summarized in Table 2. Compared with single-pollutant models, the effect estimates were slightly attenuated in two-pollutant models, but the associations generally remained significant. PM_2.5_ and PM_10_ were not mutually adjusted as they are not independent by nature. The estimates for SO_2_ and O_3_ were still non-significant in two-pollutant models. The alternative lags for temperature adjustment did not appreciably change the associations between AF and air pollutants (Supplementary Fig. 6). Similarly, we only observed very mild changes to the effect estimates by using different degrees of freedom (*df*) for temperature adjustment (Supplementary Table 4).

**Table 2:** Associations between air pollutants and atrial fibrillation in single- and two-pollutant models.

Models	PM_10_	PM_2.5_	NO_2_	SO_2_	O_3_	CO
Unadjusted	1.19 (1.03, 1.37)^a^	1.38 (1.14, 1.67)	1.60 (1.16, 2.20)	1.06 (0.84, 1.33)	0.74 (0.51, 1.06)	1.48 (1.19, 1.84)
+PM_10_	-	-	1.51 (1.09, 2.10)	1.02 (0.81, 1.29)	0.73 (0.51, 1.06)	1.43 (1.15, 1.79)
+PM_2.5_^b^	-	-	1.33 (1.04, 1.78)	0.95 (0.75, 1.20)	0.73 (0.50, 1.05)	1.22 (1.00, 1.54)
+NO_2_	1.13 (1.03, 1.28)	1.26 (1.03, 1.54)	-	0.94 (0.74, 1.20)	0.90 (0.61, 1.33)	1.30 (1.02, 1.67)
+SO_2_	1.17 (1.02, 1.35)	1.36 (1.12, 1.65)	1.54 (1.11, 2.14)	-	0.73 (0.51, 1.05)	1.44 (1.15, 1.80)
+O_3_	1.20 (1.04, 1.38)	1.40 (1.15, 1.69)	1.65 (1.18, 2.31)	1.07 (0.85, 1.35)	-	1.51 (1.21, 1.89)
+CO	1.12 (1.02, 1.29)	1.29 (1.04, 1.60)	1.44 (1.11, 1.94)	0.98 (0.77, 1.24)	0.79 (0.55, 1.14)	-

*PM*_10_ particulate matter with an aerodynamic diameter less than or equal to 10 μm, *PM*_2.5_, particulate matter with an aerodynamic diameter less than or equal to 2.5 μm, *NO*_2_ nitrogen dioxide, *SO*_2_, sulfur dioxide, *O*_3_, ozone, *CO* carbon monoxide.

^a^The associations were presented as the odds ratio of atrial fibrillation associated with each interquartile range increase (IQR) in air pollutant concentrations. The IQRs for pollutants at case hours were 45.3 μg/m^3^ for PM_10_, 25.8 μg/m^3^ for PM_2.5_, 22.0 μg/m^3^ for NO_2_, 5.8 μg/m^3^ for SO_2_, 40.6 μg/m^3^ for O_3_, and 0.3 mg/m^3^ for CO.

^b^PM_2.5_ and PM_10_ were not mutually adjusted as they are not independent by nature.

## Discussion

In this nationwide case-crossover study, we provide robust evidence that hourly exposure to ambient PM_10_, PM_2.5_, NO_2_, and CO (but not SO_2_ and O_3_) can significantly increase the risk of AF onset. By virtue of real-time AF screening from smart devices, we are able to examine the impact of air pollutants on the onset of AF episodes at the hourly level, and we identify a lag of 18–24 h as the critical time window for the effects of air pollution exposure. The exposure-response relationship curves for the four pollutants are almost linear without any discernible thresholds. The associations are also robust to the adjustment of co-pollutants. These risks are larger among females, older people, and during cool seasons.

Although previous studies have examined the acute effects of air pollutants on AF incidence, the results are generally mixed, especially for various air pollutants. For example, a meta-analysis found statistically significant associations between increased risk of AF and per unit increase in PM_2.5_ [0.89% (95%CI: 0.20%, 1.57%)], NO_2_ [1.19% (95%CI: 0.70%, 1.67%), and CO [0.60% (95%CI: 0.20%,1.09%)]^14^. Fang et al. conducted a time-series study in Yancheng, China; they found a 10 μg/m^3^ increase in PM_2.5_ was associated with a 2.81% (95%CI: 1.44%, 4.20%) change in AF hospitalization, and the estimate was 1.67% (95%CI: 0.77%, 2.59%) for PM_10_, 4.90% (95%CI: 1.69%, 8.22%) for NO_2_, and 2.55% (95%CI: 0.91%, 4.21%) for CO (per 0.1 mg/m^3^)^15^. However, in Bunch et al.’s case-crossover study in Utah, a null association between AF hospitalization and PM_2.5_ exposure was found^16^. In the present study, the effects of air pollutants (per IQR increase) on AF were strongest for NO_2_ (OR = 1.60, 95%CI: 1.16, 2.20), followed by CO (OR = 1.48, 95%CI: 1.19, 1.84), PM_2.5_ (OR = 1.38, 95%CI: 1.14, 1.67) and PM_10_ (OR = 1.19, 95%CI: 1.03, 1.37); whereas the effects for SO_2_ and O_3_ were not significant. The heterogeneity in these findings may be due to the differences in population susceptibility, sample size, air pollution levels, exposure patterns, statistical models, and outcome assessment. Besides, the present study adopted the case-crossover design using individual cases, which has a greater capacity for causal inference. More importantly, contrary to previous studies, our study could capture the exact timing of AF onsets and included paroxysmal AF events. AF is an instant cardiac arrhythmia or one-time electrical disorder within a very short time. Thus, emergency-room visits or hospitalization behavior are likely to miss the critical time window of AF onset. In the current study, we utilized a smart device-based screening technology to detect all AF episodes in real-time. In this way, we were able to explore the associations more comprehensively between air pollution and AF.

Prior research using daily hospital records of AF have reported different lag patterns in association with air pollution exposure. For example, a time-series study in Shanghai reported the largest effects of PM_2.5_ on AF hospitalization on lag 0–2 days, while another similar study in Beijing found the largest effect on lag 0–1 day^17,18^. Fang et al. reported significant associations between six air pollutants and AF hospitalization in a time-series study on lag 0–4 day^15^. In a Korean study, the association of CO with AF was most significant, with a lag of three days^19^. The sub-daily lag information for the effects of air pollution on AF episode was important to establish an early-warning system, but such a pattern was rarely explored. The current analysis linked hourly concentrations of air pollutants with real-time information on AF onset. We found the risk of AF occurred immediately after exposure and peaked at a lag of 18–24 h. Our finding was comparable to a similar case-crossover study that used ambulatory 1–lead ECG- measurements to screen AF in the old population in Stockholm^20^. They identified 469 AF episodes and found significant effects of PM_10_ on lag 12–24 h, with an OR of 1.10 (95%CI: 1.01, 1.19) per 7.8 μg/m^3^ increase in PM_10_^20^. The slight difference in lag structures may be caused by heterogeneity in the study region, sample size, pollution, and population characteristics.

The subgroup analyses indicated some potential susceptible factors. First, we consistently found larger associations between AF and all air pollutants in females, which is in line with most previous studies^10,15^. It is physiologically reasonable that women have larger heart atrium and thinner ventricular wall than men^21^, making it easier to develop AF after exposure to air pollution. In addition, Chinese women were more likely to be exposed to cooking smoke than men, and there may exist certain interactions between indoor and outdoor air pollution^22^. We only observed a slightly larger effect of air pollutants on an older population, while old age is a well-established risk factor for cardiovascular diseases, including AF^18,19^. A plausible explanation for this phenomenon is that our study employed smart devices to detect AF, and the younger population naturally tends to use and wear these watches or wristbands more frequently, which increases their likelihood of being identified. According to our pilot population screening^23^, the wearing rate was highest among the 22–39 age group (54.5%), while high-risk populations were mostly identified in the age group of 65 or older (32.4%). Lastly, the risk estimates were obviously larger in the cold than in the warm seasons. Blood vessels are constricted when exposed to cold temperatures, and cardiovascular risk factors occur more frequently in cold seasons^24^, increasing the vulnerability to additional exposure to air pollution.

The associations between air pollutants (PM_10_, PM_2.5_, NO_2_, and CO) and AF episodes were biologically plausible, although the mechanisms were not yet conclusive. The pathological process of AF involves slow remodeling of the atria, leading to disturbed electrical properties and subsequent physiological arrhythmias. Short-term air pollution exposures are not very likely to directly influence the above-prolonged processes, but they may induce AF onset through other faster pathways^25^. For example, short-term air pollution exposure could increase the secretion of acute stress hormones or sympathetic hyperexcitation^26^, increasing heart rate, blood pressure, and myocardial oxygen consumption, and subsequently leading to elevated left atrial pressure and myocardial ischemia, both of which might eventually induce AF onset. Meanwhile, air pollution could also cause immediate respiratory abnormalities (such as airway spasm and inflammation)^27^, increasing pulmonary artery pressure and elevating right atrial pressure, which might also induce AF episodes. Furthermore, as cardiac arrhythmia is a major pathway in the development of cardiovascular diseases, findings of this study could provide mechanistic insights for the previous studies that linked air pollution with the risk of other cardiovascular disorders, such as stroke and sudden cardiac arrest^28–30^. Notably, we did not find an association between O_3_ and AF, which is contrary to some existing findings^15^. Although O_3_ has been reported to have a causal link with respiratory endpoints, its impact on CVDs has always been mixed, especially for short-term O_3_ exposures^31^. As a secondary air pollutant, the formation of O_3_ is dependent on photochemical reactions with precursors and ultraviolet, and all such variations, as well as the difference in exposure assessment approaches, might contribute to the heterogeneity in previous findings. Nevertheless, additional studies are warranted to ascertain the association of O_3_ with AF.

Our findings might imply certain policy implications. Firstly, we observed monotonically increasing and almost linear exposure-response relationships between four air pollutants and AF onset. This non-threshold effect suggested a need to continuously implement stringent policies for air pollution control and stricter air quality regulatory targets. Secondly, according to our results of subgroup analyses, personal protective behaviors, and public health efforts should focus on females, older populations, and exposures during cold seasons to alleviate the hazardous effects of air pollution on AF. Thirdly, our finding on the lag pattern was particularly useful in tailoring an early-warning system for vulnerable individuals to prevent AF episodes when encountering air pollution. Fourthly, our study suggests that AF monitoring based on smart devices may be a promising and feasible approach to manage cardiovascular risks in relation to air pollution, especially for patients living in an environment of high air pollution.

The present study has several major strengths. First, we employed a smart device-based screening technology to detect AF episodes in the general population with high feasibility, which could increase the detection rate of AF (especially paroxysmal or occult AF episodes) and thus allow for a more comprehensive investigation of the association between air pollution and AF. Second, we assigned hourly exposure of air pollution to the real-time onset of AF, which could facilitate the explorations on the acute effects of multiple air pollutants on AF episodes at a very fine time scale. Third, we utilized the case-crossover study design using individual cases, which had greater strength in causal inference compared with most previous studies.

Our study was still subject to some limitations. To begin with, all environmental exposure data were derived from fixed-site monitoring stations that were closest to study participants. This may introduce ecological fallacy and measurement errors from non-differential exposure misclassification^32^. However, our case-crossover study design mainly utilized the temporal variations of exposure levels rather than the spatial variations; thus, exposure errors at the spatial scale would not considerably bias our results, but merely lead to an inflation of confidence intervals for effect estimations^33^. Secondly, we did not have the ability to apply real-time 12-lead ECG for validating all AF episodes, but the current smartphone-based screening technology demonstrated a high positive predictive value (91.6%) in a previous pilot study^11^. Thirdly, although this case-crossover design could eliminate all time-invariant confounders within a month, our results may be still subject to some residual confounding from time-varying factors (such as indoor air pollution). Fourthly, the wearing rate of smart devices was not equally distributed in the study population, with a higher wearing rate in the younger age group, but the older population were usually at higher risk of AF, which may attenuate the generalizability of our results.

In summary, this nationwide case-crossover study provides compelling new evidence on the significant associations between hourly air pollutants and AF onset. Our findings reveal that even transient exposures to PM_2.5_, PM_10_, NO_2_, and CO can significantly increase the risk of AF episodes, and the exposure-response curves suggest that there are no safe thresholds for these pollutants. Moreover, we observe that certain populations, such as females, the elderly, and those exposed during the cold season, may be at greater risk of AF associated with air pollution. These insights highlight the importance of monitoring AF episodes using wearable smart devices, especially in vulnerable populations, and underscore the urgent need for policies and interventions aimed at reducing exposure to harmful air pollutants.

## Methods

### Health data

We employed a smart device-based PPG algorithm to screen AF episodes from 2018 to 2021 across China. Details of this technology has been reported in previous publications^7,11,23,34^. In brief, a wristband (Honor Band 4) or wristwatch (Huawei Watch GT, Honor Watch, Huawei Technologies Co., Ltd., Shenzhen, China) was used for AF detection with at least 14-day monitoring. In the pilot study, individuals screened with “possible AF” were further confirmed by health providers at network hospitals with clinical evaluation, electrocardiogram, or 24-h Holter monitoring. The positive predictive value of AF episodes from PPG signals was 91.6%^11^. The sensitivity and specificity of devices in detecting AF among active recording were 95.3 and 99.7%^7^. In the current study, a total of 1,889,652 AF episodes were primarily identified. We first excluded AF episodes lasting over seven consecutive days (*N* = 1,851,741) to avoid repeated counting for a single AF case when selecting cases and controls, leaving 37,911 eligible AF episodes. Then, we further excluded repeated AF episodes that occurred within one month for each individual (*N* = 25,896) to enable the selection of control days within a month according to our case-crossover study design (to avoid overlaps of case and control periods), leaving 12,015 AF episodes. Records on residential address, gender, age, and comorbidity were also acquired when available, which were linked with AF episodes by a unique and anonymized identifier. After excluding 109 individual AF episodes with missing information on gender and age, we finally included a total of 11,906 AF episodes from 2976 participants during the study period. Each AF episode was treated as a case in this case-crossover study. The flowchart of the inclusion and exclusion process was illustrated in Supplementary Fig. 1. All participants have signed electronic informed consent before participating in the surveillance. Data authorization was approved by the Central Medical Ethic Committee of Chinese People’s Liberation Army General Hospital (S2017–105–02). This study complies with the Declaration of Helsinki.

### Assessment of exposures

The geographical locations of all included participants were presented in Supplementary Fig. 2. We assigned the exposure levels of environmental factors to each participant based on the nearest fixed-site monitoring stations. The median distance between monitoring stations and study participants was 13.1 km, ranging from 0.14 to 23.7 km. Hourly concentrations of six criteria air pollutants, including PM_10_, PM_2.5_, NO_2_, SO_2_, O_3_, and CO, were obtained from the National Air Quality Monitoring System (http://106.37.208.233:20035/). We also collected data on hourly temperature and relative humidity from the National Meteorological Information Center (http://data.cma.cn).

### Statistical analysis

We adopted a time-stratified case-crossover design to investigate the associations between hourly air pollutants and AF onset. For each AF record, the case hour was defined as the hour of AF episode onset (regardless of whether this episode persists more than or less than 1 h) on a certain day, and the control hours were selected from the same hour, day-of-week, month, and year in relation to the case hour. This is a common procedure for case-crossover studies to account for the day-of-week effect, seasonality, and long-term time trend^20^.

Conditional logistic regression models were employed to analyze the data. We built separate models for various air pollutants. We explored the lag pattern for the association by fitting air pollution concentrations as moving averages during separate lag hours from the current (lag 0 h) to 72 h prior to AF onset (e.g., lag 0–6 h, lag 6–12 h, lag 12–18 h, lag 18–24 h, lag 24–36 h, lag 36–48 h, lag 48–60 h, and lag 60–72 h). Consistent with previous studies^35,36^, we a priori selected the lag interval with the largest and most significant estimate as the lag interval used in the main analysis. We used natural spline functions to control for daily mean temperature (lag 0–24 h, *df* = 6) and relative humidity (lag 0–24 h, *df* = 3). We also graphed the exposure-response relationship between air pollution and AF using the main lag interval. Lastly, we conducted stratified analyses by potential effect modifiers, including gender, age (<60 years; ≥60 years), and season (warm season, April to September; cold season, October to March). Between-group differences were compared by the *P* value of Z-statistic calculated as follows in Eq. (1).

1 $$Z - {{{\mathrm{statistic}}}} = \frac{{{\upbeta}_2 - {\upbeta}_1}}{{\sqrt {({\mathrm{SE}}_1^2 + {\mathrm{SE}}_2^2)} }}$$

Where β_2_ and β_1_ were the estimates from two strata, and SE_1_ and SE_2_ were the standard errors for β_2_ and β_1_.

We conducted three sensitivity analyses to test the robustness of our estimates. First, we fitted two-pollutant models by adjusting for co-pollutants alternatively to test the robustness of the estimated associations at the main lag. Second, we adjusted for temperature using longer lag durations (lag 0–3d, lag 0–7d, lag 0–14d, and lag 0–21d). Third, we changed the *df* of temperature from 3 to 6 in the main models.

We performed the statistical analyses using R software, implementing conditional logistic regression using the “survival” package. Estimates were presented as the ORs of AF onset and their (95%CIs associated with each IQR increase in air pollutant concentrations. The statistical tests were two-sided, and *P* values <0.05 were considered statistically significant.

### Reporting summary

Further information on research design is available in the Nature Research Reporting Summary linked to this article.

## Supplementary information

Supplementary Information

Reporting Summary

## Data Availability

Aggregated data for analysis can be made available by contacting the corresponding authors.

## Code availability

The code that supports the findings of this study are available from the corresponding author upon reasonable request. Analysis of data and all relevant figures were generated with R version 3.6.1.
