# Supplementary material for: Application of smart devices in investigating the effects of air pollution on atrial fibrillation onset
Source: NPJ Digit Med. 2023 Mar 14;6:42. doi: 10.1038/s41746-023-00788-w (PMC10015044; doi:10.1038/s41746-023-00788-w)
Supplement: Supplementary file 1 — Supplementary Information [file 41746_2023_788_MOESM1_ESM.docx]

**Supplementary information**

**Table of contents**

[Supplementary Table 1. Summary descriptive statistics on air pollutant concentrations and meteorological conditions averaged 24 hours prior to the onset hour of atrial fibrillation and its controls. 2](#_Toc123668499)

[Supplementary Table 2. Summary descriptive statistics on air pollutant concentrations and meteorological conditions at different lag intervals prior to atrial fibrillation and its controls. 3](#_Toc123668500)

[Supplementary Table 3. Spearman correlation coefficients among the air pollutants. 5](#_Toc123668501)

[Supplementary Table 4. Odds ratios of atrial fibrillation associated with an interquartile range increase in air pollutant concentrations at lag 18–24h using different degrees of freedom of temperature. 6](#_Toc123668502)

[Supplementary Figure 1. The flowchart of inclusion and exclusion of AF episodes in this study. 7](#_Toc123668503)

[Supplementary Figure 2. Location of study participants with atrial fibrillation during the study period (N=288 cities). 8](#_Toc123668504)

[Supplementary Figure 3. Odds ratios of atrial fibrillation associated with an interquartile range increase in SO_2_ and O_3_ concentrations on different lag intervals. 9](#_Toc123668505)

[Supplementary Figure 4. Exposure-response relationship curves between SO_2_ and O_3_ concentrations and atrial fibrillation. 10](#_Toc123668506)

[Supplementary Figure 5. Odds ratios of atrial fibrillation associated with an interquartile range increase in air pollutant concentrations at lag 18–24h, stratified by gender, age and season. 11](#_Toc123668507)

[Supplementary Figure 6. Odds ratios of atrial fibrillation associated with an interquartile range increase in air pollution concentrations (at lag 18–24h), with the adjustment of temperature at different lag days. 12](#_Toc123668508)

# Supplementary Table 1. Summary descriptive statistics on air pollutant concentrations and meteorological conditions averaged 24 hours prior to the onset hour of atrial fibrillation and its controls.

| **Variables** | **Event** | **Mean ± SD** | **Percentiles** | | | | |  |
| --- | --- | --- | --- | --- | --- | --- | --- | --- |
|  |  |  | **Min** | **P25** | **P50** | **P75** | **Max** | **IQR** |
| PM_10_ (μg/m^3^) | Case | 60.5 ± 40.2 | 10.9 | 32.0 | 49.7 | 77.3 | 255.2 | 45.3 |
|  | Control | 59.8 ± 40.1 | 10.3 | 31.5 | 48.5 | 75.8 | 249.3 | 44.3 |
| PM_2.5_ (μg/m^3^) | Case | 34.2 ± 25.5 | 5.2 | 17.0 | 26.7 | 42.8 | 167.7 | 25.8 |
|  | Control | 33.3 ± 24.6 | 5.0 | 16.5 | 26.2 | 41.9 | 156.2 | 25.5 |
| NO_2_ (μg/m^3^) | Case | 30.0 ± 16.2 | 5.4 | 17.5 | 26.4 | 39.5 | 83.4 | 22.0 |
|  | Control | 29.6 ± 16.1 | 5.4 | 17.2 | 26.1 | 38.9 | 84.8 | 21.7 |
| SO_2_ (μg/m^3^) | Case | 8.4 ± 5.5 | 1.6 | 4.7 | 6.9 | 10.5 | 34.9 | 5.8 |
|  | Control | 8.4 ± 5.5 | 1.6 | 4.7 | 7.0 | 10.5 | 35.9 | 5.8 |
| O_3_ (μg/m^3^) | Case | 63.1 ± 29.2 | 7.4 | 41.3 | 60.4 | 81.9 | 149.9 | 40.6 |
|  | Control | 63.5 ± 29.3 | 8.0 | 41.5 | 60.7 | 82.2 | 150.6 | 40.7 |
| CO (mg/m^3^) | Case | 0.7 ± 0.3 | 0.2 | 0.5 | 0.7 | 0.9 | 2.0 | 0.3 |
|  | Control | 0.7 ± 0.3 | 0.2 | 0.5 | 0.7 | 0.9 | 1.9 | 0.3 |
| Temperature (℃) | Case | 15.9 ± 10.0 | -15.6 | 9.4 | 17.6 | 24.1 | 31.1 | 14.7 |
|  | Control | 15.9 ± 10.1 | -16.1 | 9.4 | 17.6 | 24.2 | 31.2 | 14.8 |
| RH (%) | Case | 68.0 ± 17.2 | 23.9 | 56.5 | 70.7 | 81.1 | 98.6 | 24.6 |
|  | Control | 67.6 ± 17.3 | 22.7 | 56.0 | 70.4 | 80.6 | 98.3 | 24.6 |

Abbreviations: SD, standard deviation; PM_10_, particulate matter with an aerodynamic diameter less than or equal to 10 μm; PM_2.5_, particulate matter with an aerodynamic diameter less than or equal to 2.5 μm; NO_2_, nitrogen dioxide; SO_2_, sulfur dioxide; O_3_, ozone; CO, carbon monoxide; RH, relative humidity.

# Supplementary Table 2. Summary descriptive statistics on air pollutant concentrations and meteorological conditions at different lag intervals prior to atrial fibrillation and its controls.

| **Variables** | **Event** | **Lag 0**–**6h** | **Lag 6**–**12h** | **Lag 12**–**18h** | **Lag 18**–**24h** | **Lag 24**–**36h** | **Lag 36**–**48h** | **Lag 48**–**60h** | **Lag 60**–**72h** |
| --- | --- | --- | --- | --- | --- | --- | --- | --- | --- |
| PM_10_ (μg/m^3^) | Case | 59.4 ± 42.0 | 59.1 ± 42.8 | 60.9 ± 44.0 | 60.9 ± 44.0 | 59.8 ± 41.3 | 60.5 ± 42.4 | 59.5 ± 41.5 | 60.2 ± 41.9 |
|  | Control | 59.4 ± 42.8 | 58.6 ± 42.6 | 59.8 ± 43.4 | 59.4 ± 42.8 | 59.2 ± 41.1 | 60.2 ± 42.3 | 59.3 ± 41.5 | 60.2 ± 42.3 |
| PM_2.5_ (μg/m^3^) | Case | 33.4 ± 27.3 | 33.6 ± 27.5 | 34.6 ± 27.9 | 34.4 ± 27.8 | 33.8 ± 26.5 | 34.2 ± 27.0 | 33.4 ± 26.1 | 33.9 ± 26.3 |
|  | Control | 32.9 ± 26.5 | 32.8 ± 26.5 | 33.8 ± 27.2 | 33.4 ± 26.8 | 33.0 ± 25.7 | 33.8 ± 26.6 | 33.1 ± 25.8 | 33.9 ± 26.6 |
| NO_2_ (μg/m^3^) | Case | 29.2 ± 18.1 | 28.2 ± 17.7 | 30.6 ± 18.9 | 31.3 ± 19.7 | 28.8 ± 16.5 | 30.9 ± 18.4 | 28.8 ± 16.8 | 30.7 ± 18.2 |
|  | Control | 28.9 ± 18.0 | 28.1 ± 17.9 | 30.2 ± 18.9 | 30.7 ± 19.3 | 28.6 ± 16.5 | 30.4 ± 17.9 | 28.6 ± 16.4 | 30.5 ± 17.9 |
| SO_2_ (μg/m^3^) | Case | 8.3 ± 5.9 | 8.4 ± 5.9 | 8.4 ± 6.0 | 8.2 ± 5.9 | 8.4 ± 5.7 | 8.3 ± 5.8 | 8.5 ± 5.9 | 8.4 ± 5.8 |
|  | Control | 8.3 ± 5.9 | 8.3 ± 5.9 | 8.4 ± 6.0 | 8.1 ± 5.8 | 8.4 ± 5.7 | 8.4 ± 5.8 | 8.4 ± 5.7 | 8.3 ± 5.8 |
| O_3_ (μg/m^3^) | Case | 66.7 ± 37.8 | 66.5 ± 41.5 | 57.6 ± 35.1 | 58.8 ± 37.1 | 66.9 ± 34.0 | 58.8 ± 31.7 | 66.8 ± 34.2 | 58.9 ± 31.6 |
|  | Control | 67.2 ± 38.0 | 66.5 ± 41.5 | 58.2 ± 35.4 | 59.4 ± 37.3 | 67.1 ± 34.5 | 59.5 ± 32.0 | 66.8 ± 34.0 | 59.2 ± 31.8 |
| CO (mg/m^3^) | Case | 0.7 ± 0.3 | 0.7 ± 0.3 | 0.7 ± 0.3 | 0.7 ± 0.3 | 0.7 ± 0.3 | 0.7 ± 0.3 | 0.7 ± 0.3 | 0.7 ± 0.3 |
|  | Control | 0.7 ± 0.3 | 0.7 ± 0.3 | 0.7 ± 0.3 | 0.7 ± 0.3 | 0.7 ± 0.3 | 0.7 ± 0.3 | 0.7 ± 0.3 | 0.7 ± 0.3 |
| Temperature (℃) | Case | 15.5 ± 10.3 | 17.4 ± 10.3 | 16.1 ± 10.3 | 14.7 ± 10.4 | 16.4 ± 10.1 | 15.4 ± 10.2 | 16.4 ± 10.2 | 15.4 ± 10.3 |
|  | Control | 15.6 ± 10.3 | 17.4 ± 10.4 | 16.1 ± 10.4 | 14.7 ± 10.5 | 16.5 ± 10.2 | 15.5 ± 10.3 | 16.5 ± 10.2 | 15.5 ± 10.3 |
| RH (%) | Case | 69.7 ± 19.9 | 62.6 ± 22.1 | 68.6 ± 19.0 | 72.9 ± 20.1 | 65.7 ± 19.5 | 69.5 ± 18.5 | 65.6 ± 19.6 | 69.5 ± 18.5 |
|  | Control | 69.4 ± 19.9 | 62.2 ± 22.2 | 68.1 ± 19.2 | 72.5 ± 20.2 | 65.5 ± 19.4 | 69.4 ± 18.5 | 65.6 ± 19.4 | 69.6 ± 18.4 |

Abbreviations as in Supplementary Table 1.

# Supplementary Table 3. Spearman correlation coefficients among the air pollutants.

| Pollutants | PM_10_ | PM_2.5_ | NO_2_ | SO_2_ | CO | O_3_ | Temp | RH |
| --- | --- | --- | --- | --- | --- | --- | --- | --- |
| PM_10_ | 1.00 |  |  |  |  |  |  |  |
| PM_2.5_ | 0.84 | 1.00 |  |  |  |  |  |  |
| NO_2_ | 0.58 | 0.57 | 1.00 |  |  |  |  |  |
| SO_2_ | 0.42 | 0.34 | 0.31 | 1.00 |  |  |  |  |
| CO | 0.46 | 0.59 | 0.52 | 0.31 | 1.00 |  |  |  |
| O_3_ | -0.04 | -0.10 | -0.39 | -0.07 | -0.26 | 1.00 |  |  |
| Temp | -0.37 | -0.39 | -0.35 | -0.30 | -0.23 | 0.48 | 1.00 |  |
| RH | -0.40 | -0.17 | -0.17 | -0.24 | 0.08 | -0.17 | 0.27 | 1.00 |

Abbreviations as in Supplementary Table 1.

# Supplementary Table 4. **Odds ratios of atrial fibrillation associated with an interquartile range increase in air pollutant concentrations at lag 18–24h using different degrees of freedom of temperature.**

| **Df of temperature** | **PM_10_** | **PM_2.5_** | **NO_2_** | **SO_2_** | **O_3_** | **CO** |
| --- | --- | --- | --- | --- | --- | --- |
| Df=3 | 1.247 (1.081, 1.439) | 1.452 (1.201, 1.756) | 1.994 (1.446, 2.751) | 1.116 (0.891, 1.399) | 0.777 (0.539, 1.119) | 1.557 (1.255, 1.933) |
| Df=4 | 1.248 (1.081, 1.439) | 1.452 (1.201, 1.756) | 1.991 (1.443, 2.746) | 1.115 (0.890, 1.398) | 0.778 (0.541, 1.121) | 1.557 (1.255, 1.933) |
| Df=5 | 1.250 (1.083, 1.442) | 1.455 (1.203, 1.760) | 1.994 (1.445, 2.752) | 1.114 (0.889, 1.397) | 0.778 (0.541, 1.121) | 1.559 (1.256, 1.935) |
| Df=6 ^1^ | 1.249 (1.083, 1.441) | 1.455 (1.203, 1.760) | 1.995 (1.446, 2.753) | 1.114 (0.889, 1.396) | 0.778 (0.540, 1.120) | 1.559 (1.256, 1.936) |

Abbreviations: Df, degree of freedom.

Notes: ^1^ temperature with a degree of freedom of 6 was used in the main analysis.


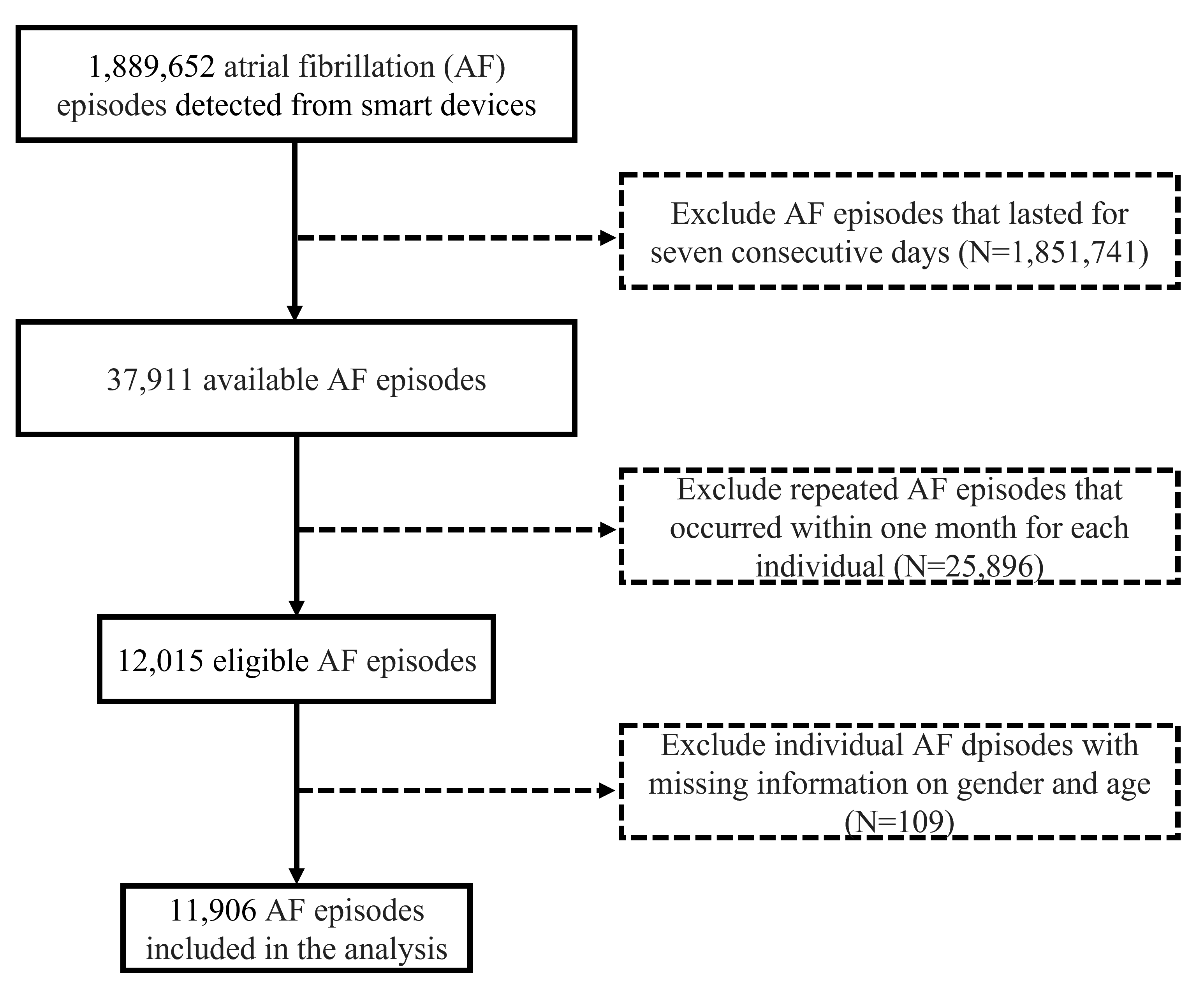


# Supplementary Figure 1. The flowchart of inclusion and exclusion of AF episodes in this study.


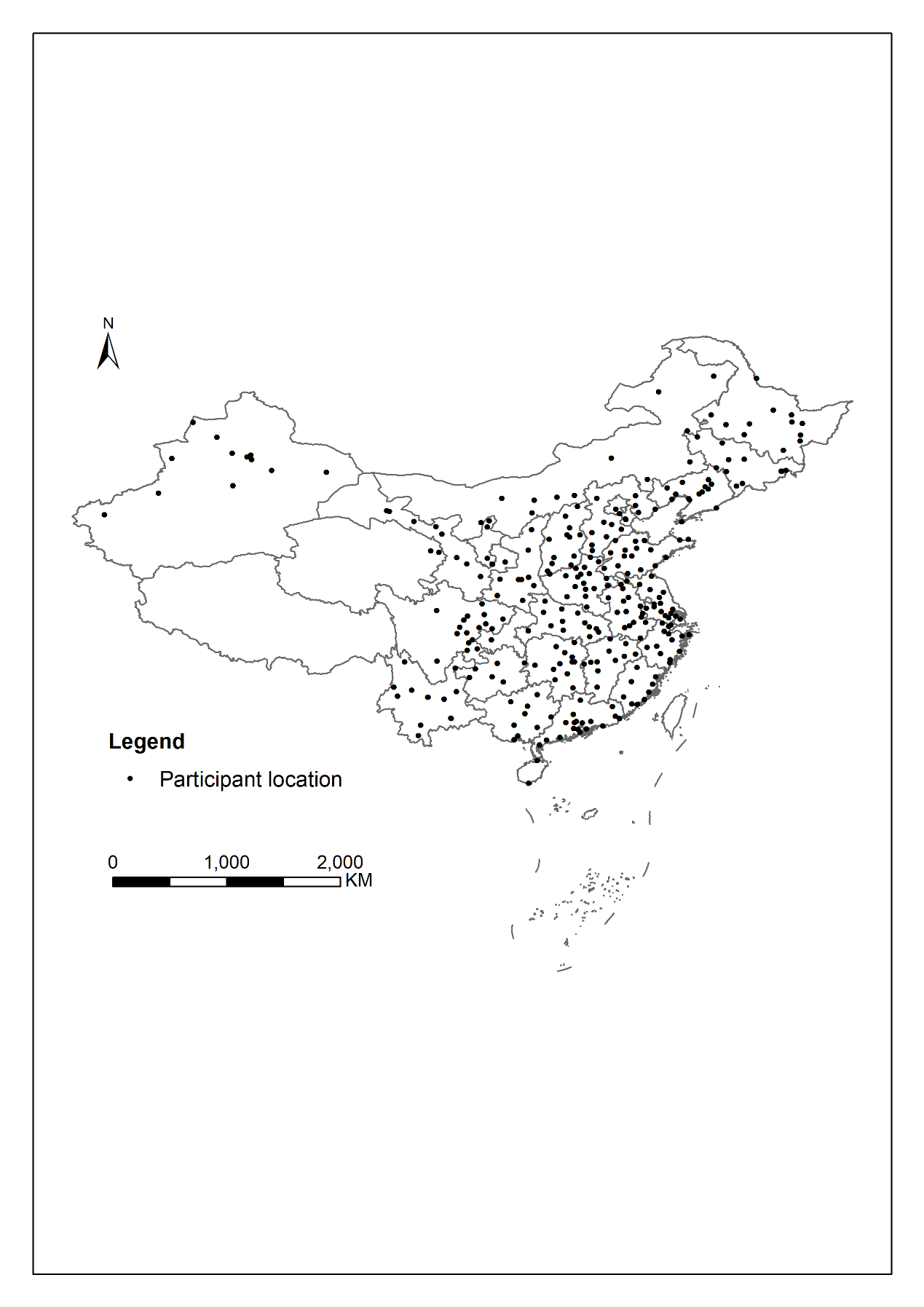


# Supplementary Figure 2. Location of study participants with atrial fibrillation during the study period (N=288 cities).


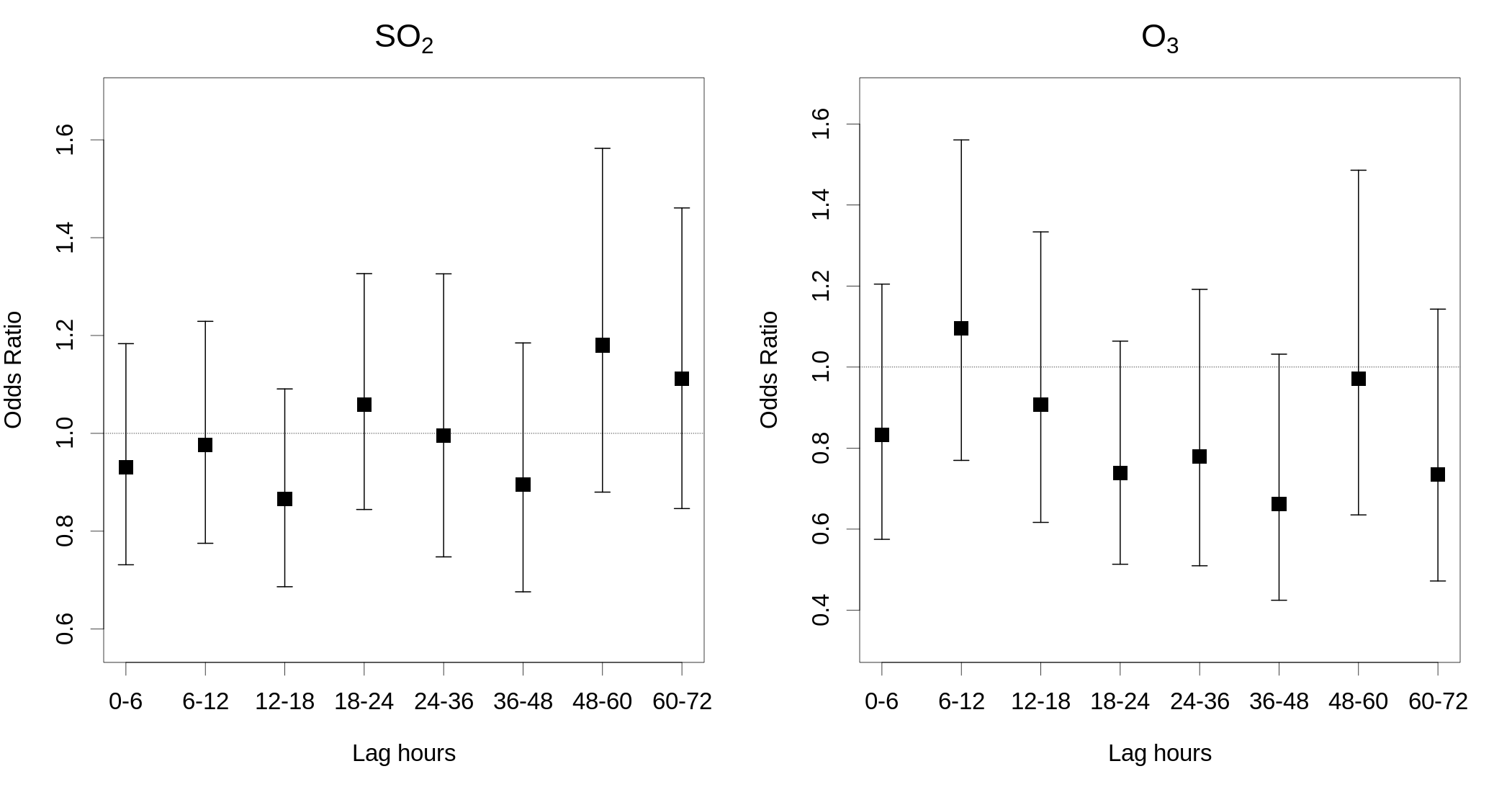


# Supplementary Figure **3. Odds ratios of atrial fibrillation associated with an interquartile range increase in SO_2_ and O_3_ concentrations on different lag intervals.**

Abbreviations and interquartile range concentrations as in Supplementary Table 1. Lags hours, e.g., Lag 0–6, the moving average concentrations of the current to the previous 6 hours; Lag 6–12, the moving average concentrations of the previous 7 to the previous 12 hours. Error bars are defined as standard deviation (s.d.).


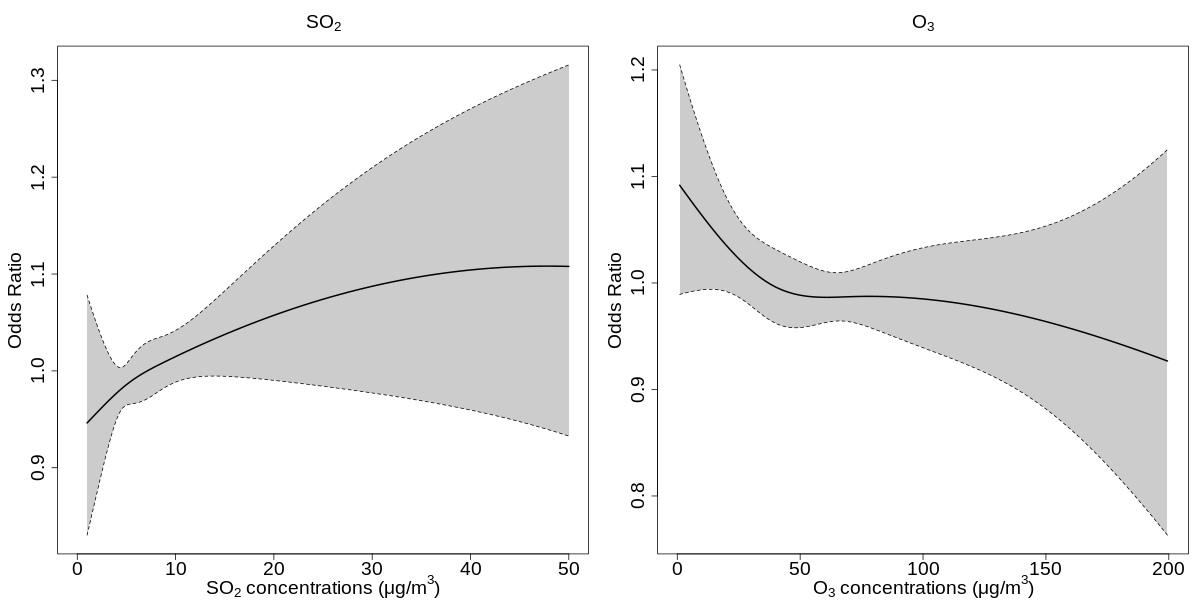


# Supplementary Figure **4. Exposure-response relationship curves between SO_2_ and O_3_ concentrations and atrial fibrillation.**

The associations were presented as the odds ratio of atrial fibrillation associated with each unit increase in air pollutant concentrations at lag 18–24h. The black lines were mean estimates and the shaded areas were 95% confidence intervals. Abbreviations as in Supplementary Table 1.


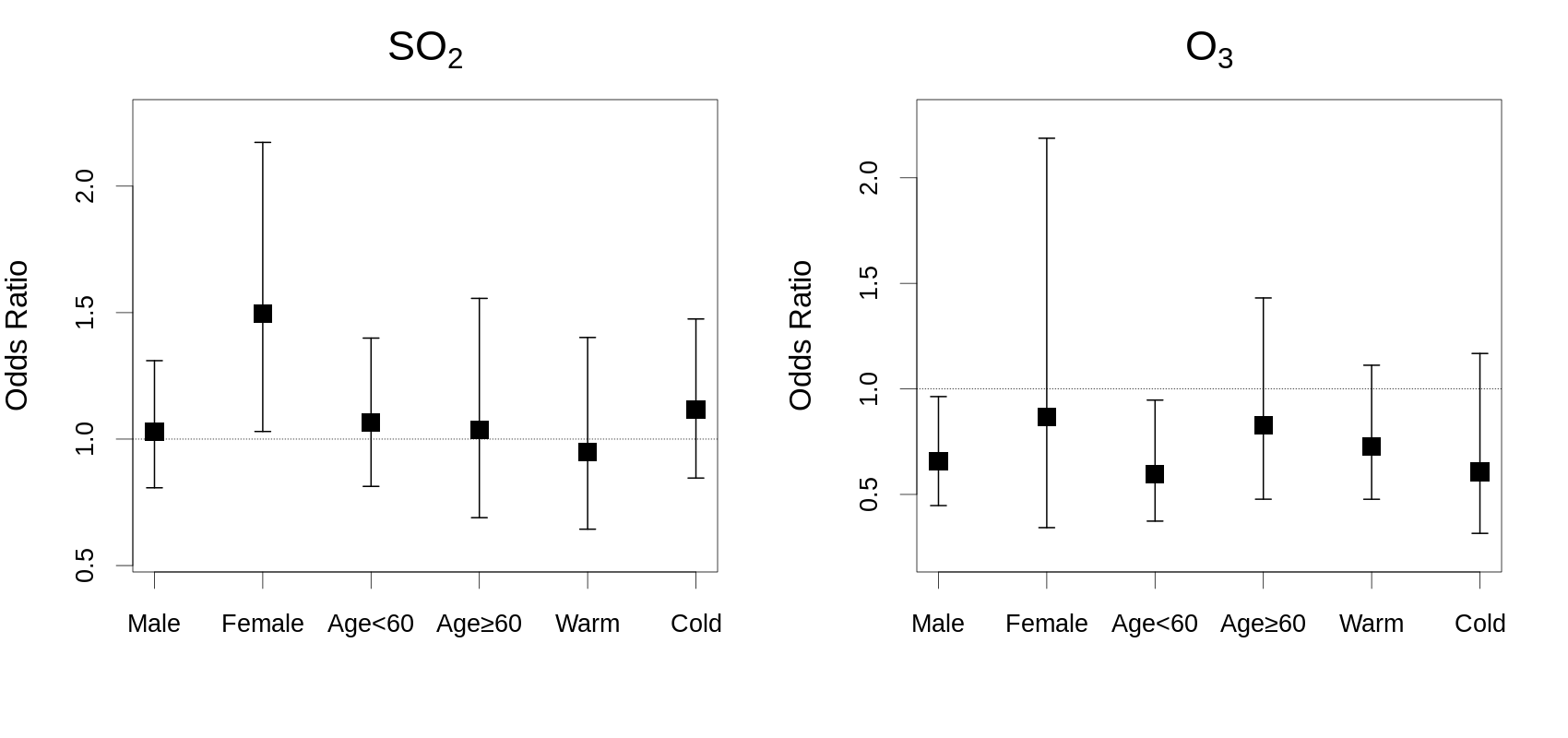


# Supplementary Figure **5. Odds ratios of atrial fibrillation associated with an interquartile range increase in air pollutant concentrations at lag 18–24h, stratified by gender, age and season.**

Abbreviations and interquartile range concentrations as in Supplementary Table 1. Warm season, April to September; Cold season, October to March. Error bars are defined as standard deviation (s.d.).


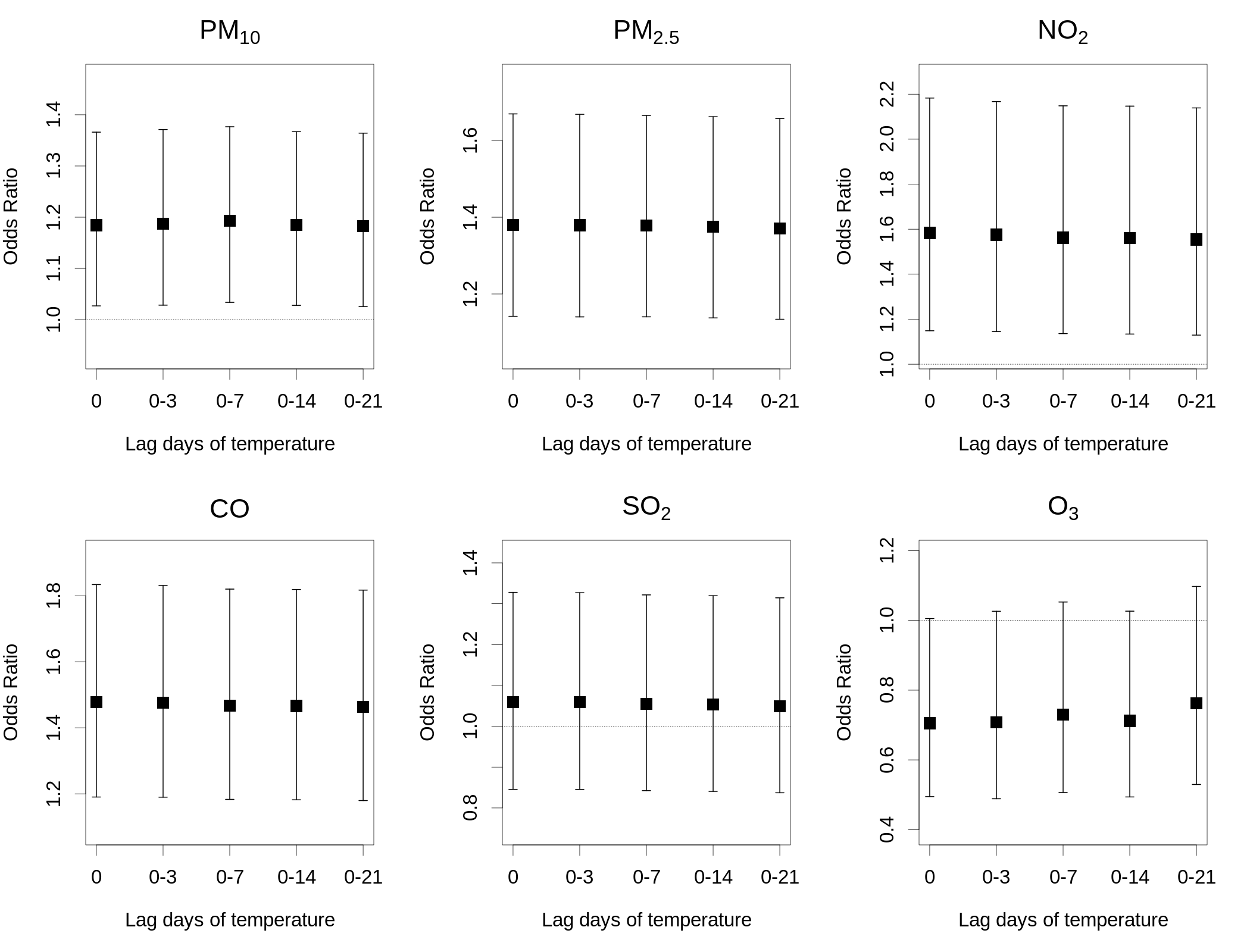


# Supplementary Figure 6**. Odds ratios of atrial fibrillation associated with an interquartile range increase in air pollution concentrations (at lag 18–24h), with the adjustment of temperature at different lag days.**

Abbreviations and interquartile range concentrations as in Supplementary Table 1. Lag days of temperature, e.g., Lag 0–3, the moving average temperature of the current to the previous three days. Error bars are defined as standard deviation (s.d.).
