# Supplementary material for: Application of smart devices in investigating the effects of air pollution on atrial fibrillation onset
Source: NPJ Digit Med. 2023 Mar 14;6:42. doi: 10.1038/s41746-023-00788-w (PMC10015044; doi:10.1038/s41746-023-00788-w)
Supplement: Supplementary file 2 — Reporting Summary [file 41746_2023_788_MOESM2_ESM.pdf]

nature portfolio

Corresponding author(s): Renjie Chen

Last updated by author(s): Feb 22, 2023

Reporting Summary

Nature Portfolio wishes to improve the reproducibility of the work that we publish. This form provides structure for consistency and transparency in reporting. For further information on Nature Portfolio policies, see our Editorial Policies and the Editorial Policy Checklist.

Statistics

For all statistical analyses, confirm that the following items are present in the figure legend, table legend, main text, or Methods section.

n/a	Confirmed	
☐	☒	The exact sample size (n) for each experimental group/condition, given as a discrete number and unit of measurement
☒	☐	A statement on whether measurements were taken from distinct samples or whether the same sample was measured repeatedly
☐	☒	The statistical test(s) used AND whether they are one- or two-sided<br>*Only common tests should be described solely by name; describe more complex techniques in the Methods section.*
☐	☒	A description of all covariates tested
☐	☒	A description of any assumptions or corrections, such as tests of normality and adjustment for multiple comparisons
☐	☒	A full description of the statistical parameters including central tendency (e.g. means) or other basic estimates (e.g. regression coefficient) AND variation (e.g. standard deviation) or associated estimates of uncertainty (e.g. confidence intervals)
☐	☒	For null hypothesis testing, the test statistic (e.g. F, t, r) with confidence intervals, effect sizes, degrees of freedom and P value noted<br>*Give P values as exact values whenever suitable.*
☒	☐	For Bayesian analysis, information on the choice of priors and Markov chain Monte Carlo settings
☐	☒	For hierarchical and complex designs, identification of the appropriate level for tests and full reporting of outcomes
☐	☒	Estimates of effect sizes (e.g. Cohen's d, Pearson's r), indicating how they were calculated

Our web collection on statistics for biologists contains articles on many of the points above.

Software and code

Policy information about availability of computer code

Data collection	We employed a smart device–based PPG algorithm to screen atrial fibrillation episodes in the general population from 2018 to 2021 across China.
Data analysis	We performed the statistical analyses using R software, implementing conditional logistic regression using the "survival" package.

For manuscripts utilizing custom algorithms or software that are central to the research but not yet described in published literature, software must be made available to editors and reviewers. We strongly encourage code deposition in a community repository (e.g. GitHub). See the Nature Portfolio guidelines for submitting code & software for further information.

Data

Policy information about availability of data

All manuscripts must include a data availability statement. This statement should provide the following information, where applicable:

- Accession codes, unique identifiers, or web links for publicly available datasets
- A description of any restrictions on data availability
- For clinical datasets or third party data, please ensure that the statement adheres to our policy

Aggregated data for analysis can be made available by contacting the corresponding authors.

Human research participants

Policy information about studies involving human research participants and Sex and Gender in Research.

Reporting on sex and gender	For AF episodes, males accounted for a significantly larger proportion (85.9%) than females (14.1%).
Population characteristics	We identified a total of 11,906 AF episodes with 40,551 controls (3.4/1) in 2,976 participants during the study period from 2018 to 2021, covering 288 cities in China. There were more cases in warm season (54.8%) compared with cold season (45.2%). Notably, our study observed more AF episodes in individuals with age < 60 years (59.7%)
Recruitment	We employed a smart device–based PPG algorithm to screen AF episodes from 2018 to 2021 across China. In brief, a wristband (Honor Band 4) or wristwatch (Huawei Watch GT, Honor Watch, Huawei Technologies Co., Ltd., Shenzhen, China) was used for AF detection with at least 14-day monitoring. In the current study, a total of 1,889,652 AF episodes were primarily identified. We firstly excluded AF episodes lasting over seven consecutive days (N=1,851,741) to avoid repeated counting for a single AF case when selecting case and controls, leaving 37,911 eligible AF episodes. Then, we further excluded repeated AF episodes that occurred within one month for each individual (N=25,896) to enable the selection of control days within a month according to our case-crossover study design (to avoid overlaps of case and control periods), leaving 12,015 AF episodes. Records on residential address, gender, age, and comorbidity were also acquired when available, which were linked with AF episodes by a unique and anonymized identifier. After excluding 109 individual AF episodes with missing information on gender and age, we finally included a total of 11,906 AF episodes from 2,976 participants during the study period.
Ethics oversight	All participants have signed electronic informed consent before participating in the surveillance. Data authorization was approved by the Central Medical Ethic Committee of Chinese People's Liberation Army General Hospital (S2017-105-02). This study complies with the Declaration of Helsinki.

Note that full information on the approval of the study protocol must also be provided in the manuscript.

Field-specific reporting

Please select the one below that is the best fit for your research. If you are not sure, read the appropriate sections before making your selection.

☐ Life sciences ☐ Behavioural & social sciences ☒ Ecological, evolutionary & environmental sciences

For a reference copy of the document with all sections, see nature.com/documents/nr-reporting-summary-flat.pdf

Ecological, evolutionary & environmental sciences study design

All studies must disclose on these points even when the disclosure is negative.

Study description	We adopted a time-stratified case-crossover design to investigate the associations between hourly air pollutants and AF onset.
Research sample	We finally included a total of 11,906 AF episodes from 2,976 participants during the study period. Each AF episode was treated as a case in this case-crossover study
Sampling strategy	A wristband or wristwatch was used for AF detection with at least 14-day monitoring.
Data collection	We employed a smart device–based PPG algorithm to screen atrial fibrillation episodes in the general population from 2018 to 2021 across China.
Timing and spatial scale	We employed a smart device–based PPG algorithm to screen AF episodes from 2018 to 2021 across China.
Data exclusions	In the current study, a total of 1,889,652 AF episodes were primarily identified. We firstly excluded AF episodes lasting over seven consecutive days (N=1,851,741) to avoid repeated counting for a single AF case when selecting case and controls, leaving 37,911 eligible AF episodes. Then, we further excluded repeated AF episodes that occurred within one month for each individual (N=25,896) to enable the selection of control days within a month according to our case-crossover study design (to avoid overlaps of case and control periods), leaving 12,015 AF episodes. Records on residential address, gender, age, and comorbidity were also acquired when available, which were linked with AF episodes by a unique and anonymized identifier. After excluding 109 individual AF episodes with missing information on gender and age, we finally included a total of 11,906 AF episodes from 2,976 participants during the study period.
Reproducibility	We conducted three sensitivity analyses to test the robustness of our estimates. First, we fitted two-pollutant models by adjusting for co-pollutants alternatively to test the robustness of the estimated associations at the main lag. Second, we adjusted for temperature using longer lag durations (lag 0–3d, lag 0–7d, lag 0–14d, and lag 0–21d). Third, we changed the df of temperature from 3 to 6 in the main models.
Randomization	Not applicable.

Blinding	Not applicable.

Did the study involve field work? ☐ Yes ☒ No

Reporting for specific materials, systems and methods

We require information from authors about some types of materials, experimental systems and methods used in many studies. Here, indicate whether each material, system or method listed is relevant to your study. If you are not sure if a list item applies to your research, read the appropriate section before selecting a response.

Materials & experimental systems

n/a	Involved in the study
☒	☐ Antibodies
☒	☐ Eukaryotic cell lines
☒	☐ Palaeontology and archaeology
☒	☐ Animals and other organisms
☒	☐ Clinical data
☒	☐ Dual use research of concern

Methods

n/a	Involved in the study
☒	☐ ChIP-seq
☒	☐ Flow cytometry
☒	☐ MRI-based neuroimaging
